# The role of peroxisome proliferator-activated receptors in the modulation of hyperinflammation induced by SARS-CoV-2 infection: A perspective for COVID-19 therapy

**DOI:** 10.3389/fimmu.2023.1127358

**Published:** 2023-02-17

**Authors:** Aliakbar Hasankhani, Abolfazl Bahrami, Bahareh Tavakoli-Far, Setare Iranshahi, Farnaz Ghaemi, Majid Reza Akbarizadeh, Ali H. Amin, Bahman Abedi Kiasari, Alireza Mohammadzadeh Shabestari

**Affiliations:** ^1^ Department of Animal Science, College of Agriculture and Natural Resources, University of Tehran, Karaj, Iran; ^2^ Faculty of Agricultural Sciences and Engineering, University of Tehran, Karaj, Iran; ^3^ Dietary Supplements and Probiotic Research Center, Alborz University of Medical Sciences, Karaj, Iran; ^4^ Department of Physiology and Pharmacology, School of Medicine, Alborz University of Medical Sciences, Karaj, Iran; ^5^ School of Pharmacy, Shahid Beheshty University of Medical Sciences, Tehran, Iran; ^6^ Department of Biochemistry, Faculty of Advanced Sciences and Technology, Tehran Medical Sciences, Islamic Azad University, Tehran, Iran; ^7^ Department of Pediatric, School of Medicine, Amir al momenin Hospital, Zabol University of Medical Sciences, Zabol, Iran; ^8^ Zoology Department, Faculty of Science, Mansoura University, Mansoura, Egypt; ^9^ Virology Department, Faculty of Veterinary Medicine, University of Tehran, Tehran, Iran; ^10^ Department of Dental Surgery, Mashhad University of Medical Sciences, Mashhad, Iran; ^11^ Khorasan Covid-19 Scientific Committee, Mashhad, Iran

**Keywords:** SARS-CoV-2, cytokine storm, PPARs, hyperinflammation, COVID-19 therapy,

## Abstract

Coronavirus disease 2019 (COVID-19) is a severe respiratory disease caused by infection with severe acute respiratory syndrome coronavirus 2 (SARS-CoV-2) that affects the lower and upper respiratory tract in humans. SARS-CoV-2 infection is associated with the induction of a cascade of uncontrolled inflammatory responses in the host, ultimately leading to hyperinflammation or cytokine storm. Indeed, cytokine storm is a hallmark of SARS-CoV-2 immunopathogenesis, directly related to the severity of the disease and mortality in COVID-19 patients. Considering the lack of any definitive treatment for COVID-19, targeting key inflammatory factors to regulate the inflammatory response in COVID-19 patients could be a fundamental step to developing effective therapeutic strategies against SARS-CoV-2 infection. Currently, in addition to well-defined metabolic actions, especially lipid metabolism and glucose utilization, there is growing evidence of a central role of the ligand-dependent nuclear receptors and peroxisome proliferator-activated receptors (PPARs) including PPARα, PPARβ/δ, and PPARγ in the control of inflammatory signals in various human inflammatory diseases. This makes them attractive targets for developing therapeutic approaches to control/suppress the hyperinflammatory response in patients with severe COVID-19. In this review, we (1) investigate the anti-inflammatory mechanisms mediated by PPARs and their ligands during SARS-CoV-2 infection, and (2) on the basis of the recent literature, highlight the importance of PPAR subtypes for the development of promising therapeutic approaches against the cytokine storm in severe COVID-19 patients.

## 1 Introduction

Coronavirus disease 2019 (COVID-19) is an infectious and severe respiratory disease caused by severe acute respiratory syndrome coronavirus 2 (SARS-CoV-2). SARS-CoV-2 is a positive sense single-stranded RNA beta-coronavirus that infects the lower and upper respiratory tract and has recently affected millions of people worldwide (1–3). Primary symptoms of COVID-19 include fever, cough, pneumonia, and shortness of breath, and histological pictures of this disease are characterized by mononuclear inflammatory cells, severe pneumocyte hyperplasia, interstitial thickening, hyaline membrane formation, and prominent alveolar damage with eosinophilic exudates (4, 5).

During COVID-19, a cascade of inflammatory pathways is activated, leading to massive cytokine release from the host immune system in response to SARS-CoV-2 infection (6, 7). In this regard, the vast increase in the secretion of circulating proinflammatory cytokines such as tumor necrosis factors (TNFs), interleukins (ILs), chemokines, and interferons (IFNs) leads to the exacerbation of the host inflammatory response to the pathogen. This exacerbation in the host’s inflammatory response increases the severity of the disease (8–10). This hyperinflammation or imbalanced inflammation during SARS-CoV-2 infection is called “cytokine storm”, which is one of the main hallmarks of the deterioration of the COVID-19 immunopathogenesis and triggers acute respiratory distress syndrome (ARDS), multi-organ failure (MOF), acute lung injury (ALI), decreased lung function, and finally, death of the host (11, 12). In this context, in recent years, various clinical and omics-based studies have investigated the molecular mechanisms behind the SARS-CoV-2 infection in different disease stages and different tissues (13–17). Surprisingly, most of these studies have observed the activation of inflammatory mechanisms and hyperinflammation in COVID-19 patients. Therefore, developing effective therapeutic strategies by targeting critical factors in regulating host inflammatory response can provide a potential and promising solution for the survival of COVID-19 patients, especially the prevention of cytokine storms (18–20). The peroxisome proliferator-activated receptors (PPARs) are a subgroup of ligand-activated transcription factors and members of the nuclear receptor superfamily that play a crucial role in regulating energy balance, carbohydrate and lipid metabolism, cell growth, and differentiation (21, 22). PPARs can regulate the transcriptional activity of target genes by two different mechanisms (1): binding to the promoter region of target genes with DNA sequences known as peroxisome proliferator response elements (PPREs) as a ligand-dependent transcription factor, and (2) controlling gene expression through association with PPRE-independent activator proteins (21, 23). Several previous reports highlighted the core role of PPARs in many human diseases, such as different types of cancer (24, 25), atherosclerosis (26), and type 2 diabetes (27, 28).

Interestingly, in addition to the central roles of PPARs in regulating energy homeostasis, such as fatty-acid metabolism and glucose utilization, growing evidence suggests that members of the nuclear receptor superfamily, such as PPARs, also have significant regulatory effects on inflammatory processes (29). Indeed, extensive research has proven that PPARs have potential anti-inflammatory effects during inflammation-related disease (30, 31). In this regard, previous literature, based on available evidence, has suggested that subtypes of PPARs exert their anti-inflammatory effects and subsequently control the host’s inflammatory response through different mechanisms such as successful competition with other inflammatory transcription factors for the recruitment of essential and shared co-activator proteins, inhibition of binding of inflammatory transcription factors such as AP-1, nuclear factor-κB (NF-κB), NFAT, and STATs to their response elements through direct physical protein-protein interaction, blocking MAPK-induced signaling cascades, preventing the clearance of proinflammatory genes co-repressors, and upregulation in the expression of anti-inflammatory genes (32). For example, it has been discussed that the activation of PPARs during inflammatory bowel disease (IBD) leads to the suppression of the main pathways of inflammation, such as NF-κB signaling. Subsequently, the activation of PPARs inhibits the production of proinflammatory cytokines such as TNF-α, IL6, and IL1B. Therefore, it was concluded that anti-inflammatory responses induced by the activation of PPARs might restore the physio-pathological imbalance associated with this disorder (21).

The interference of viral infections such as SARS-CoV-2 in the PPARs signaling is a completely new issue and interest in this area has been very motivated by the COVID-19 pandemic. Emerging studies show that SARS-CoV-2, by modulating PPAR subtypes, leads to metabolic changes (especially lipid metabolism) and exacerbation of pulmonary inflammation in lung epithelial cells of COVID-19 patients (33). Therefore, these findings have suggested that the use of agonists of PPARs with the aim of their activation may be a useful therapeutic strategy to reverse the inflammatory and metabolic changes caused by SARS-CoV-2 infection (34). In this regard, it has been reported that several natural ligands of PPARs, such as turmeric, docosahexaenoic acid (DHA), and eicosapentaenoic acid (EPA), lead to a decrease in the production of proinflammatory cytokines through interaction with PPARs and then induction of their activity (35, 36) For example, a very recent study has identified possible mechanisms by which the PPARα agonist palmitoylethanolamide (PEA) antagonizes the NF-κB signaling pathway and subsequently reduces the production of TNF-α, IL1B, and other inflammatory mediators such as inducible nitric oxide synthase (iNOS) and COX2 through selective activation of PPARα in cultured murine alveolar macrophages during SARS-CoV-2 infection (37). Moreover, it has been suggested that synthetic agonists of PPARγ, such as thiazolidinediones (TZDs), like pioglitazone, are anti-inflammatory drugs with ameliorative effects on severe viral pneumonia-like COVID-19 (38). On the other hand, by integrating different transcriptome datasets with computational network-based systems biology methods, promising therapeutic targets, including PPARα and PPARγ, have been identified for the modulation of inflammatory processes caused by COVID-19 (39, 40). Therefore, PPARs and their ligands have crucial therapeutic potential with key immunomodulatory effects on inflammatory mechanisms and cytokine/chemokine production during infectious and inflammation-related diseases such as the COVID-19 pandemic.

Nevertheless, considering the importance of immunopathology’s role of the inflammatory response in COVID-19 patients and the role of PPARs in controlling inflammation, in this review, we (1) provide a summary of general information about PPARs such as subtypes, structure, tissue expression, and function (2), investigate the molecular mechanisms of the exacerbation of the host inflammatory response during COVID-19 (3), describe the anti-inflammatory mechanisms mediated by PPARs, and (4) discuss the anti-inflammatory roles of PPAR subtypes during COVID-19 pandemic on the basis of the recent clinical and omics-based literature.

## 2 PPARs: Subtypes, structure, tissue expression, and function

### 2.1 PPAR subtypes and structure

**Figure 1:**
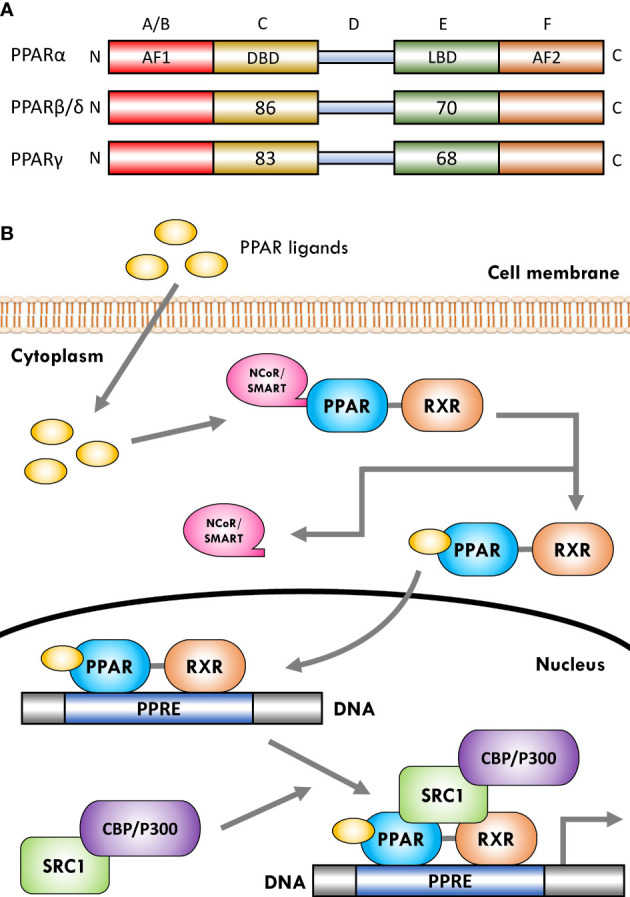
Schematic representation of peroxisome proliferator-activated receptor (PPAR) structure and ligand-induced activation. **(A)** The PPARs are composed of five distinct regions or domains (1): the ligand-independent activation domain of AF1 located in the N-terminus (amino-terminal A/B domain), which is responsible for receptor phosphorylation (2); the highly conserved DNA-binding domain **(DBD)** in the C region that contains two zinc finger motifs responsible for receptor binding to DNA targets on the peroxisome proliferator hormone response elements (PPREs) of PPARs target genes (3); a variable hinge region in the D domain that is the docking site for co-factors (4); a moderately conserved ligand-binding domain (LBD) in the E region that is responsible for the ligand specificity and activation, as well as for dimerization of the receptor with the 9-cis-retinoic acid receptor (RXR) (5); an AF2 ligand-dependent activation domain in the C-terminus (carboxyl-terminal in F domain) that is crucial for the recruitment of PPAR co-activators. Numbers shown in C and E regions indicate the percentage amino-acid identity of DBD and LBD of human PPARβ/δ and PPARγ compared to human PPARα. **(B)** Several co-activator or co-repressor factors affect the activity of PPARs, which can stimulate or inhibit the function of the receptor, respectively. When PPARs are in a non-ligand-bound state in solution (inactive mode), all three PPAR isoforms can bind transcription co-repressors in a DNA-independent manner. These co-repressors, such as nuclear receptor co-repressor/silencing mediators for retinoid and thyroid hormone receptors (NCoR/SMRT), suppress gene transcription by interacting with histone deacetylases (HDACs). The binding of ligands to the PPAR–RXR heterodimer causes the exchange of co-repressors with co-activators, thereby converting PPARs from an inactive state to an active state. Receptor activation generally occurs after agonist binding to the LBD. Following ligand binding and initiation of receptor phosphorylation, PPARs dissociate the co-repressor complex. Then, the ligand–heterodimer (ligand–PPAR–RXR) complex binds to the target DNA promoter through a PPRE. Next, in order to allow the transcriptional machinery to gain access to the promoter region, PPARs bind specific co-activator complexes, such as steroid receptor activator 1 (SRC1) and cAMP response element-binding (CREB)-binding protein (CBP)/p300, which have acetyltransferase activity. Subsequently, they regulate the transcription of various genes that play a key role in various physiological processes.

The PPARs are ligand-dependent/activated transcription factors, members of the nuclear- hormone-receptor superfamily (including the receptors for thyroid hormone, vitamin D, ecdysone, retinoic acids, and some orphan receptors) that transduce a wide range of signals, including environmental, nutritional, and inflammatory events, to a set of cellular responses at the transcriptional gene level; they were named due to their joint property in increasing the number and activity of peroxisomes (41–43). So far, three isoforms of PPARs, namely, PPARα (NR1C1), PPARβ/δ (NR1C2), and PPARγ (NR1C3), have been identified in vertebrates (including human, mouse, rat, hamster, and Xenopus), which are encoded by distinct genes on different chromosomes. They have shown a high degree of sequence and structural homology (**Figure 1A**) but different tissue distribution, ligand specificity, and regulatory activities (44–46).

### 2.2 Tissue distribution and function

In recent years, various *in vitro* and *in vivo* studies have reported that all isoforms of PPARs primarily regulate lipid and glucose metabolism and have additional regulatory roles in cell proliferation and differentiation, vascular homeostasis and atherosclerosis, cancer, and the immune system (38, 47). In addition to the mentioned activities, it is thought that the activation of PPAR subtypes reduces the expression of proinflammatory cytokines and inflammatory cell functions, exerting significant anti-inflammatory properties (48). PPARα is the first known PPAR that was initially cloned from a mouse liver complementary DNA library as a nuclear receptor that mediates the effects of an endogenous group and xenobiotic compounds known as peroxisome proliferators (PPs) (31, 49). This subtype of PPARs is highly expressed in metabolically active tissues such as the liver, heart, skeletal muscles, intestinal mucosa, and brown adipose tissue (50, 51). PPARα is mainly involved in the carbohydrate metabolism and catabolism of fatty acids and their oxidation, such that its activation reduces lipid levels (52–55). Additionally, it has been well highlighted that PPARα increases the expression of IκB, which is a factor that suppresses the nuclear translocation and transcriptional activity of NF-κB, thereby interfering with NF-κB signaling and the inflammatory response (48). Besides, increasing evidence has demonstrated that the anti-inflammatory properties of PPARα are manifested by a decrease in the secretion of several key downstream inflammatory factors such as NF-κB–driven cytokines (TNF-α, IL1B, and IL6), COX2, IL8, IL12, IL2, VCAM1, TLR4, MCP1, STAT3, AP-1, and IL18 (56, 57). Moreover, it has been reported that the activation of PPARα leads to the upregulation of important anti-inflammatory factors such as IL1 receptor antagonist (IL1ra) (58) and vanin-1 (59). Furthermore, PPARα can interfere with angiogenic responses that are critical during chronic inflammation by targeting endothelial vascular endothelial growth factor receptor-2 (VEGFR-2) signaling, thereby controlling the inflammatory response (60).

PPARγ is the most widely studied PPAR isoform, which is expressed in white and brown adipose tissue, large intestine, and immune cells such as macrophages, the pancreas, and the spleen, and it plays a key role in a series of biochemical processes, including insulin sensitivity, inducing tumor cell differentiation and apoptosis, adipogenesis, lipoprotein metabolism, energy balance, reducing blood fat and blood pressure, and lipid biosynthesis (53, 61–63). Activation of PPARγ increases fat storage by increasing adipocyte differentiation and enhancing the transcription of genes important for lipogenesis (64, 65). Moreover, this subtype has been proposed as a potential therapeutic target for different types of cancer due to its various anti-tumor properties (66, 67). In terms of regulating inflammation, recent literature has reported that PPARγ prevent the inflammatory cascades caused by NF-κB activation and the production of proinflammatory cytokines such as TNF-α, IL1B, IFN-γ, IL2, iNOS, IL18, reactive oxygen species (ROS), and IL6 through the inhibition of NF-κB transactivation (38, 68–70). On the other hand, PPARγ exerts its protective effects by targeting major inflammatory factors such as STAT1, AP-1, PI3K, intercellular adhesion molecule (ICAM1), and matrix metallopeptidase 9 (MMP9) and inhibiting their activity to prevent destructive inflammatory damage (71). In addition, PPARγ regulates the expression of several essential inflammatory target genes such as MCP1/CCL2, endothelin-1, and adiponectin (APN) (72). Interestingly, a recent study well demonstrated that PPARγ inhibits dysregulated inflammatory responses by suppressing NLRP3 inflammasome activation as well as decreasing maturation of caspase-1 and IL1B (73).

The PPARβ/δ is the third subtype of PPARs, which has not been as intensely studied as PPARα and PPARγ; it consists of 441 amino acids with a molecular weight of 49.9 kDa. This isoform is expressed in almost all tissues. It is especially abundant in the liver, intestine, kidney, abdominal adipose tissue, and skeletal muscle, all of which are involved in lipid metabolism. Indeed, the PPARβ/δ isoform participates in fatty-acid oxidation, mainly in skeletal and cardiac muscles, and regulates blood cholesterol and glucose concentration (47, 74, 75). However, complete information on the exact role of PPARβ/δ in the regulation of inflammation is still not available, and more research is needed to deeply dissect the relationship between PPARβ/δ and inflammation or inflammatory response. In some contexts, PPARβ/δ has been shown to have anti-inflammatory functions. For example, it was demonstrated that activation of PPARβ/δ reduces the expression of inflammation-associated NF-κB and STAT1-targeted genes including TNF-α, MCP1, IL6, CXCL8, CCL2, CXCR2, and CXCL1 (76–79). Taken together, all three PPAR subtypes have distinct yet overlapping roles in regulating metabolic function and inflammation. Further details on the tissue distribution, function, and natural and synthetic ligands of PPARα, PPARβ/δ, and PPARγ are provided in **Table 1**.

**Table 1:** The natural and synthetic ligands, tissue expression, and function of PPARs.

PPAR Subtypes	Main Ligands (Natural and Synthetic)	Function	Tissue Distribution	Reference
PPARα (NR1C1)	Unsaturated fatty acids, omega-3, leukotriene B4, 8-hydroxy-eicosatetraenoic acid, clofibrate, fenofibrate, gemfibrozil, bezafibrate, and ciprofibrate	Lipid catabolism and hemostasis by stimulating beta-oxidation of fatty acids, control of inflammatory processes and vascular integrity, and mediation of the hypolipidemic function of fibrates	Highly expressed in metabolically active tissues such as liver, heart, kidney, large intestine, skeletal muscle, intestinal mucosa, and brown adipose	(22, 47, 53, 54, 80)
PPARβ/δ (NR1C2)	Arachidonic acid, linoleic acid, PGI213s, 13S-HODE, carbaprostacyclin, components of VLDL, GW501516, GW0742, MBX-802, and L-165041	Responsible for glucose metabolism and homeostasis, vascular integrity, glycogen metabolism, and control of inflammation	Expressed ubiquitously in virtually all tissues, mostly expressed in the small intestine and large intestine, and highly expressed in skin, skeletal muscle, adipose tissue, inflammatory cells, and heart	(81–84)
PPARγ (NR1C3)	Unsaturated fatty acids, prostaglandin PGJ2, 15-hydroxy-eicosatetraenoic acid, 9- and 13-hydroxy-octadecadienoic acid, 15-deoxyΔ12,14-prostaglandin G2, prostaglandin PGJ2, ciglitazone, pioglitazone, rosiglitazone, troglitazone, farglitazar, S26948, and INT131	Lipid storage, glucose disposal, insulin sensitivity, cellular proliferation, differentiation, regulation of innate immune response and inflammation, and differentiation and maturation of adipocytes	Expressed at the highest level in adipose tissue (white and brown), as well as in epithelial surfaces, urinary tract, human placental trophoblast, immunologic system (bone marrow, lymphocytes, monocytes, and macrophages), and spleen	(61, 62, 85–88)

### 2.3 Mechanism of PPAR activation

The activation of PPARs by ligands is associated with structural changes in the receptor, including dissociation from co-repressor complexes and association with appropriate transcriptional co-activators, binding to DNA, and acquiring transactivation/transrepression capabilities (31). Moreover, promotion of many biochemical mechanisms of PPARs requires that the receptor is part of a heterodimeric complex with another nuclear receptor, the 9-cis-retinoic acid receptor (RXR; NR2B) (21, 89). Therefore, after activation with ligands/agonists, the PPAR–RXR heterodimers are transported to the nucleus and bind to specific DNA sequences consisting of a direct repeat of DNA recognition motif AGGTCA separated by one or two nucleotides (DR-1 or DR-2 response elements), thereby stimulating/repressing the transcription of target genes (**Figure 1B**) (89, 90). This sequence is called the peroxisome proliferator response element (PPRE) and is located in the promoter regions of PPAR-regulated target genes (91). Furthermore, after binding the ligand-activated PPAR–RXR complex to the target DNA through PPARE, this complex binds to specific co-activator complexes such as CREB-binding protein (CBP)/p300 and steroid receptor co-activator 1 (SRC1), which have histone acetyltransferase activity and facilitate the remodeling of chromatin structure (92–95). In this regard, previous studies have reported that the binding of co-activator complexes to the ligand-activated, PPRE-associated PPAR–RXR complex can disrupt nucleosomes and induce transcriptional regulatory changes in the chromatin structure near the regulatory regions of PPAR target genes (**Figure 1B**) (57, 96, 97).

## 3 COVID-19 and cytokine storm

**Figure 2:**
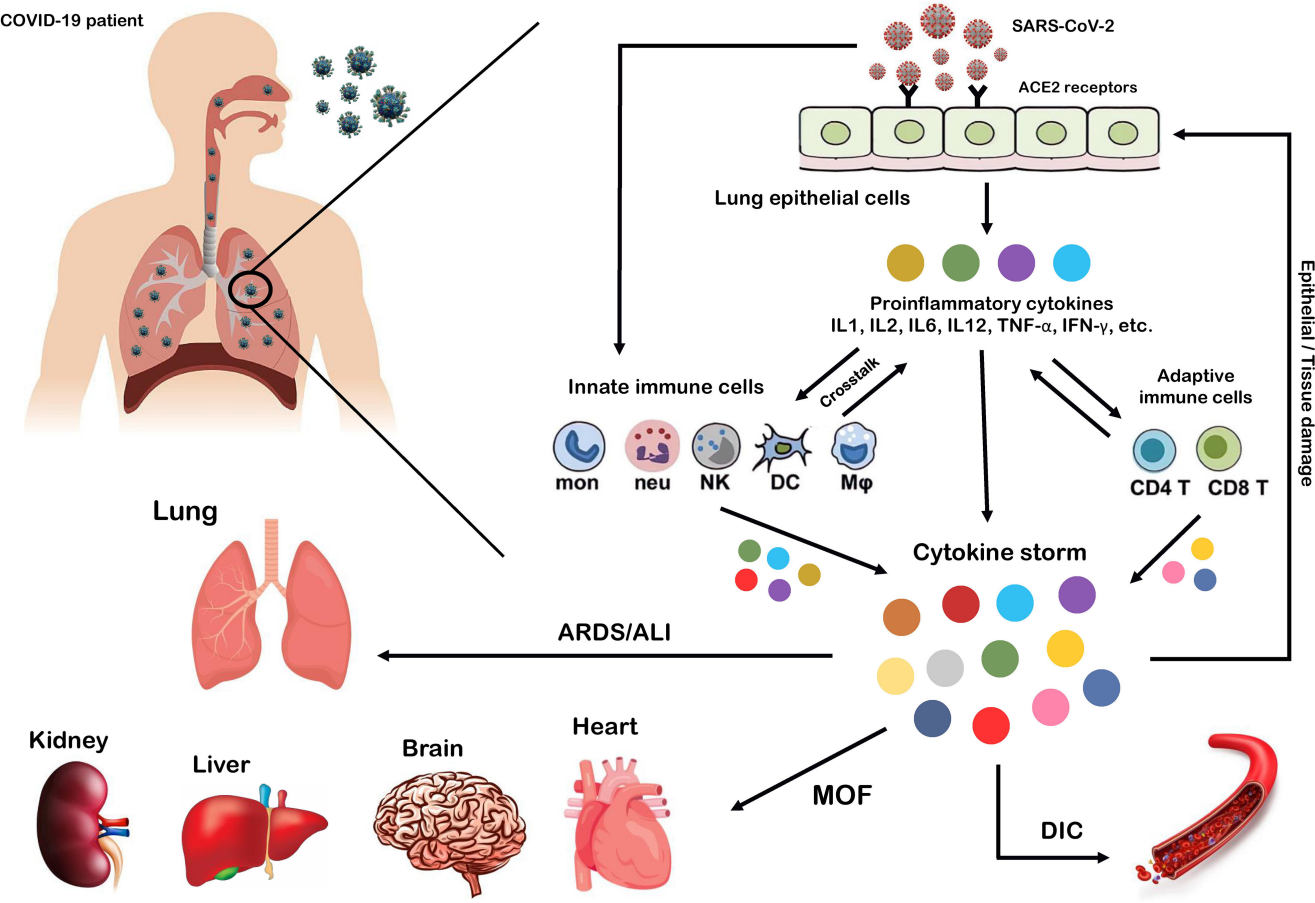
Cytokine storm as the hallmark of COVID-19 immunopathogenesis. Following the entry of SARS-CoV-2 into lung epithelial and immune cells *via* angiotensin-converting enzyme 2 (ACE2) receptors, a cascade of downstream signaling pathways is activated, ultimately leading to the massive release of proinflammatory cytokines and chemokines and tissue damage. Moreover, these proinflammatory cytokines lead to the recruitment of more innate immune cells, including neutrophils, macrophages, natural killer (NK) cells, monocytes, and dendritic cells (DCs) and active adaptive immune cells, including CD4+ and CD8+ T cells, to the site of infection, in order to induce the production of circulating cytokines. As a result, the crosstalk between epithelial and immune cells in vast cytokine release causes hyperinflammation and cytokine storm, which leads to a wide range of clinical manifestations from mild to severe/critical forms with a fatal outcome. Some of these fatal consequences include macrophage activation syndrome (MAS), hemophagocytic lymphohistiocytosis (HLH), capillary leak syndrome (CLS), thrombosis, disseminated intravascular coagulation (DIC), acute respiratory distress syndrome (ARDS), multi-organ failure (MOF), and acute lung injury (ALI).

SARS-CoV-2, which affects the lower and upper respiratory tract, invades host cells through angiotensin-converting enzyme 2 (ACE2) receptors (98, 99) and causes a wide range of clinical manifestations from mild forms such as fever, cough, and myalgia to moderate forms with pneumonia and local inflammation symptoms requiring hospitalization, to severe/critical forms with fatal outcomes (100). Upon cellular entry of SARS-CoV-2 *via* its ACE2 receptor, viral genomic single-stranded RNA or other RNA compositions (double-stranded RNA) can be recognized as pathogen-associated molecule patterns (PAMPs) by innate immune and epithelial cells through the activation of pattern recognition receptors (PRRs) such as Toll-like receptors (TLRs), retinoic acid-inducible gene-I (RIG-I)-like receptors (RLRs), and NOD-like receptors (NLRs) (101, 102). Following sensitization of PRRs, downstream key inflammation-related transcription factors such as NF-κB, activator protein-1 (AP-1), and IFN regulatory factors (IRFs) are activated and promote the transcription of proinflammatory cytokines, chemokines, and IFNs such as IL1β, IL18, IL6, IL12, TNF-α, IL8, IL2, IL7, IL17, CCL3, CCL5, CXCL8, CXCL10, and IFN-γ (103–108). Moreover, proinflammatory cytokines such as IL6, TNF-α, and IFN-γ, in turn, activate JAK/STAT, NF-κB, and mitogen-activated protein kinase (MAPK) signaling by binding to their receptors on immune cells to induce further production of proinflammatory cytokines and subsequently form positive feedback to initiate the cytokine storm (**Figure 2**) (102).

Exacerbation of the local inflammatory response and increased secretion of proinflammatory cytokines and chemokines by resident immune and respiratory epithelial cells leads to more recruitment of innate and adaptive immune cells such as macrophages, neutrophils, dendritic cells (DCs), natural killer (NK) cells, monocytes, and CD4+ and CD8+ T cells to the site of infection to produce more persistent inflammatory cytokines (102). Indeed, growing evidence suggests that the crosstalk between epithelial cells and immune cells in COVID-19 produces high levels of proinflammatory cytokines that trigger an uncontrollable inflammatory response, hyperinflammation, or imbalanced inflammation (known as “cytokine storm”) with severe complications and poor outcomes (109, 110). In this regard, extensive studies have recently reported that high circulating levels of proinflammatory cytokines (IFN-α, IFN-γ, IL1β, IL6, IL12, IL18, IL33, TNF-α, TGF-β, IL1RA, IL7, IL8, IL9, VEGFA, etc.) and chemokines (CCL2, CCL3, CCL5, CXCL8, CXCL9, CXCL10, etc.) have been identified in patients with severe COVID-19 (105, 111–114). Furthermore, it has been highlighted that cytokine storm is one of the main features of ARDS, ALI, tissue damage, and MOF, which are the major causes of COVID-19 severity and death of patients (106, 111, 115). Therefore, we believe that any intervention approach to target the critical inflammatory factors during SARS-CoV-2 infection could be a fundamental step in developing therapeutic strategies to control hyperinflammation, combat the cytokine storm, and reduce COVID-19 severity.

## 4 Anti-inflammatory mechanisms mediated by PPARs

In the last decade, many studies have concluded that PPARs, in addition to being critical players in glucose and lipid metabolism, play an essential role in controlling various types of the inflammatory response (57, 116, 117). Indeed, the inflammatory role of PPARs was highlighted when a previous study showed that PPARα knockdown was directly associated with increased levels of proinflammatory cytokines (118). In agreement with this study, a recent study showed that, in addition to PPARα, the knockdown of PPARγ also leads to increased serum levels of IL6, IL1β, and TNF-α during lipopolysaccharide (LPS) stimulation (119). Furthermore, in an animal model, Huang et al. (120) showed that increased PPARγ expression levels prevented pulmonary inflammation and were directly associated with the recovery of influenza virus-infected animals (120). Moreover, several previous studies have shown that PPARα and PPARγ activation lead to reduced inflammation in polymicrobial sepsis (121) and HIV infection (122). In addition to these findings, in recent years, the central role of PPARs to control inflammation and reduce the levels of proinflammatory cytokines has been reviewed in many inflammatory disorders, including lung inflammatory diseases (32), IBD (21), and hepatic inflammation (116). These results indicate that PPARs suppress the transcription of main active inflammatory transcription factors, including NF-κB, AP-1, nuclear factor of activated T cells (NFAT), and signal transducers and activators of transcription (STATs), through an agonist-dependent mechanism (123). Among the various mechanisms PPARs use to repress many distinct transcriptions factor families, the most likely include four main mechanisms in which ligand-activated PPAR–RXR complexes suppress the activity of many inflammatory factors.

**Figure 3:**
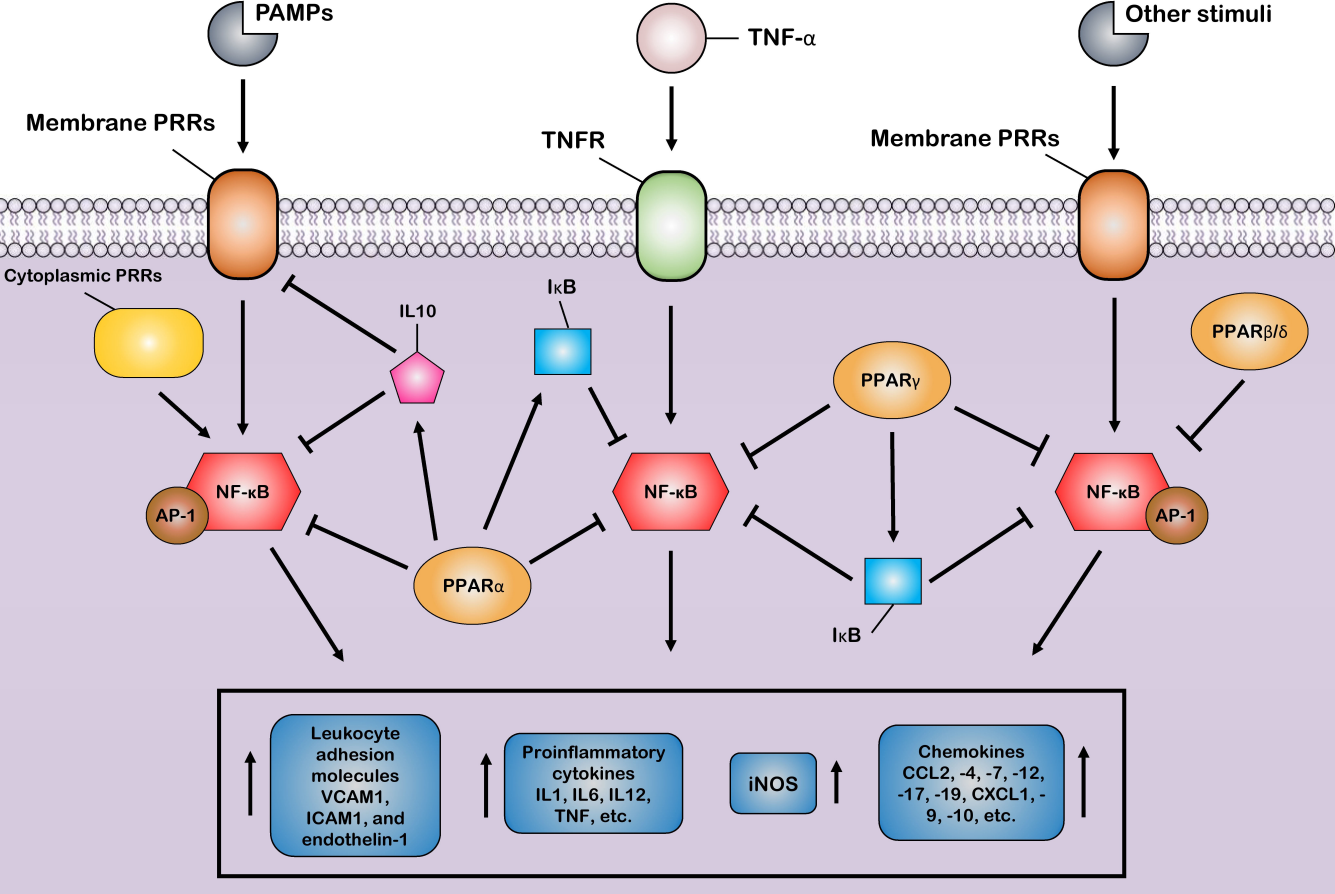
Control of the host inflammatory response mediated by PPARs. Most of the anti-inflammatory properties of PPARs are characterized by the suppression of key inflammatory transcription factors such as nuclear factor-κB (NF-κB) and activator protein-1 (AP-1) *via* different suppressive mechanisms, as induction of the production of anti-inflammatory cytokines. Through these mechanisms, PPARs block the expression of various inflammatory genes and, thus, reduce the production of many proinflammatory cytokines, chemokines, and other proinflammatory signal mediators, such as inducible nitric oxide synthase (iNOS). Additionally, the PPARs prevent the recruitment of leukocytes to the site of inflammation by inhibiting the production of cell adhesion molecules.

The first mechanism is the successful competition of PPARs to limit the amount of essential and shared co-activator proteins (such as CBP/P300) in a cell. As a result of this successful competition, these co-activators are not available for other transcription factors (31, 124). Therefore, the activities of other transcription factors (such as NF-κB) that use the same co-activators are repressed in these situations of co-activator competition. On the other hand, the second mechanism involves direct physical association between PPARs and other transcription factors without the mediation of co-activators. During the second mechanism, known as “cross-coupling” or “mutual receptor antagonism”, ligand-activated PPAR–RXR heterodimers form a new complex with other transcription factors, such as AP-1, NF-κB, NFAT, and STATs through physical protein–protein interactions, thereby preventing transcription factor binding to its response element and also inhibiting their ability to induce the transcription of proinflammatory genes such as IL6, IL1β, and TNF-α (**Figure 3**) (46). For instance, agonist-activated PPARα and PPARγ negatively regulate the inflammatory gene response through bidirectionally blocking NF-κB and AP-1 signaling pathways *via* physical interaction with NF-κB p65 (38, 125). Moreover, PPARs can suppress the expression of NF-κB through the upregulation of inhibitors of NF-κB (IκBs) (126, 127). The third mechanism also involves blocking MAPK-inducted signaling cascades by ligand-activated PPAR–RXR heterodimers through inhibition of MAPK phosphorylation and activation (128, 129). Lastly, preventing the clearance of co-repressors whose removal is required for the transcriptional activation of AP-1 and NF-κB target proinflammatory genes is the fourth mechanism of inflammation suppression by PPARs (130, 131). Moreover, another anti-inflammatory effect of PPARs is their agonistic effect with other anti-inflammatory factors. Previous studies have shown that a significant increase in the expression level of PPARs is associated with an increase in the expression of anti-inflammatory factors such as IL10 (132–134). Several human and animal models have reported that PPARs and their ligands downregulate the expression of many chemokines such as CCL2, -4, -7, -12, -17, and -19, CXCL1, -9, and -10, and leukocyte adhesion molecules such as VCAM1, ICAM1, and endothelin-1. This downmodulation inhibits leukocyte recruitment to the site of inflammation and reduces the crosstalk between immune cells and other resident cells for cytokine production (135–139).

## 5 Control of inflammation by PPARs during SARS-CoV-2 infection

The recent emergence of COVID-19 in the past years and its rapid worldwide spread have led to extensive clinical studies investigating the molecular regulatory mechanisms behind this severe disease. Intriguingly, at this time, many of these extensive clinical studies reported the influential role of PPAR subtypes, especially PPARγ and their ligands, in controlling the host inflammatory response during SARS-CoV-2 infection. Meanwhile, in previous clinical trials, a decrease in the expression of PPAR subtypes and an increase in the serum level of proinflammatory cytokines have been observed in inflammatory lungs of patients with severe COVID-19 (112, 113, 140). Additionally, in agreement with the results of clinical studies, several recent transcriptomics studies using microarray, RNA-sequencing (RNA-seq), and single-cell RNA-seq techniques have reported downregulation of PPARs in various tissues including whole blood, lung epithelial cells, bronchoalveolar lavage fluids (BALFs), and peripheral blood mononuclear cells (PBMCs) in the SARS-CoV-2-infected individuals (141–145). Following these findings, the results of previous proteomics and metabolomics studies also indicate the interference of SARS-CoV-2 infection in PPAR signaling (146, 147). Surprisingly, Keikha et al. (148) recently demonstrated that a set of miRNAs, including mir-27b, were upregulated during SARS-CoV-2 infection. They also reported that mir-27b has a significant negative correlation with its main target, i.e., PPARγ, and the increase in its expression during SARS-CoV-2 infection directly leads to the downregulation of PPARγ, thus playing a key role in the exacerbation of the inflammatory response in COVID-19 patients (148). One of the main immunopathogenesis strategies of SARS-CoV-2 infection has been suggested to interfere with PPAR signaling to exacerbate the inflammatory response (149). In other words, previous studies have reported that the decrease in the expression of PPARs including PPARγ, PPARα, and PPARβ/δ during SARS-CoV-2 infection is associated with the increased secretion of proinflammatory cytokines such as IL6, IL1β, and TNF-α, as well as cytokine storm; thus, it has a positive correlation with ARDS and ALI in COVID-19 patients (150, 151).

Moreover, a previous study concluded that SARS-CoV-2 suppresses PPAR expression in the lungs and abrogates one of the main anti-inflammatory cores for NF-κB activity, thereby exerting a hyperinflammatory response in patients with severe COVID-19 (152). Moreover, several recent studies also reported that the reduction in PPARγ and PPARα is directly related to acute pulmonary inflammation in COVID-19 and the shift of the disease from mild to severe and, finally, death (33, 153). Additionally, it has been highlighted that suppressing the expression of PPAR subtypes, especially PPARγ, leads to increased susceptibility to SARS-CoV-2 infection (154). Interestingly, the decrease in PPARγ expression during SARS-CoV-2 infection, in addition to being positively related to the occurrence of hyperinflammation, also leads to insulin resistance in COVID-19 patients (155). Besides, it has recently been reviewed that over-activation of the canonical WNT/β-catenin pathway in response to SARS-CoV-2 infection leads to inhibition of PPARγ expression in an opposing interplay (156). Furthermore, COVID-19 has more negative clinical consequences for obese people because clinical trials indicate that the serum levels of PPARγ are lower in obese people. Therefore, the probability of cytokine storm during SARS-CoV-2 infection is higher in these people (157). Furthermore, another study proved that alcohol consumption is directly related to systematic inflammation in COVID-19 patients because ethanol (EtOH) exacerbates the activation of proinflammatory cytokines, including IL6, IL1B, IFN, and TNF-α and inflammation-related transcription factors, including HIF1-α, JUN, NF-κB, and STATs *via* induction of PPAR–RXR inactivation (158).

Additionally, there is accumulating evidence that the T-helper 2 (Th2) inflammatory response phenotype can induce protective effects against the COVID-19 immunopathogenesis due to the increased secretion and release of Th2 anti-inflammatory cytokines such as IL10, IL4, and IL13 and recruitment of the eosinophils to the site of inflammation (159). Following these results, it has been well reviewed that cytokines associated with Th2 inflammatory response such as IL4 and IL13 inhibit the secretion of several proinflammatory cytokines such as IL6, IL1B, IL1α, IL12, and TNF-α, which play a central role in the pathogenesis of COVID-19 and hyperinflammation (160). Moreover, the anti-inflammatory M2 macrophages is activated by IL4 and IL13, which modulate inflammatory responses by producing anti-inflammatory cytokines, such as IL10 (161). Strikingly, recent results suggest that Th2 responses which driven by IL4, IL5, and IL13 dramatically reduce ACE2 in the respiratory tract and are associated with better clinical outcomes with COVID-19 (162, 163). Therefore, it has been hypothesized that the Th2 inflammatory response may exert potential protective effects against COVID-19 (164). Surprisingly, previous studies in several human inflammatory diseases indicate that both PPARα and PPARγ and their ligands increase the expression levels of anti-inflammatory markers associated with the Th2 inflammation such as IL13, IL4, IL10, and GATA3, thereby limiting the dysregulation of inflammation (165–167). Therefore, based on these findings, it can be concluded that PPARs can induce different anti-inflammatory mechanisms during SARS-CoV-2 infection through a synergistic effect with Th2 inflammatory responses.

Several reports suggest that PPARs play an important role in controlling the inflammatory response during COVID-19 by inducing the inactivation of the key inflammatory transcription factors, especially NF-κB (168). In this regard, it has been suggested that activation of PPARγ during COVID-19 can reduce the circulating levels of TNF-α, IL-1, and IL-6 in the innate immune cells such as macrophages and monocytes through interaction with NF-κB (169). Moreover, PPARγ acts as a negative regulator of cytotoxic T-cell activation and suppresses the production of cytokines by these adaptive immune cells (170). Following these studies, the recent emerging literature has also reported that activation of PPARα, PPARβ/δ, and PPARγ is inversely related to pulmonary fibrosis caused by chronic inflammation in COVID-19 patients (147, 171–173). It has been also hypothesized that exercise may prevent untoward systemic consequences of SARS-CoV-2 infection including inflammation and metabolic dysfunctions such as lipotoxicity by having a positive effect on PPARα (33). The anti-inflammatory role of PPARβ/δ during COVID-19 has been less studied than the other two types of PPARs. However, a few reports have indicated that PPARβ/δ suppressing transcription factors involved in the inflammatory response, including NF-κB and AP-1 (31), reduce the expression levels of GDF-15 (one of the inflammatory biomarkers of COVID-19 severity) in a negative feedback manner (174).

Furthermore, PPARβ/δ and PPARγ have been shown to play a central role in the macrophage polarization toward an anti-inflammatory M2 phenotype during COVID-19 (175). On the other hand, the previous literature has shown that PPARα and PPARγ prevent the apoptosis of inflammatory cells by inducing anti-apoptotic factors of the BCL-2 family, thereby preventing the spread of cytokines and chemokines in the intercellular space (176). Notably, the decrease in the expression levels of PPARs in the early stages of SARS-CoV-2 infection and the increase in their expression during the treatment/recovery period indicate the opposite/inverse relationship of these receptors with the severity of the disease (38, 177).

Intriguingly, PPARα and PPARγ have been proposed as effective adjuvants for the development of COVID-19 vaccines because these receptors through an increase in the population of regulatory T-cells *via* upregulation in FOXP3 mRNA expression (a transcriptional factor for the function and differentiation of regulatory T-cells) (1): stimulate memory T-cells (2), upregulate the γδ type of T-cells, and (3) prolong B-cell memory and improve the secondary antibody response and thus can induce long-term memory (176, 178). However, the inverse relationship between regulatory T-cells and chronic inflammation has been revealed by previous research (175, 179), and the anti-inflammatory properties of these cells are well established (180, 181). Therefore, it can be predicted that the increase in the population of regulatory T-cells due to the activation of PPARs can play a potential dual-role by stimulating and strengthening long-term memory and exerting significant anti-inflammatory properties during SARS-CoV-2 infection.

Recent advances in high-throughput transcriptome-based technologies and the integration of these techniques with computational network-based algorithms of systems biology have provided an excellent opportunity to identify altered gene regulatory networks under infected conditions, activated pathways, potential therapeutic/diagnostic/prognostic targets, and understanding the complex molecular mechanisms underlying infectious disease at the systemic level (182). In our previous work, we integrated and analyzed the RNA-seq data from PBMCs of healthy individuals and COVID-19 patients with computational network-based methods of systems biology in order to identify potential therapeutic targets and candidate gene modules underlying COVID-19 and develop promising therapeutic strategies for COVID-19 (39). As a result, we identified nine candidate co-expressed gene modules and 290 hub-high traffic genes with the highest betweenness centrality (BC) score directly related to SARS-CoV-2 pathogenesis (39). Indeed, the genes with the highest BC score have the highest rate of “information transfer” in their respective modules with critical biological functions, which are known as “high traffic” genes and can be potential therapeutic, diagnostic, and prognostic targets for COVID-19 therapy (39). We observed that PPARα is among the hub-high traffic genes in one of the key modules with anti-inflammatory function, indicating the crucial anti-inflammatory role of this PPAR subtype during SARS-CoV-2 infection (39). In another study, Auwul et al. (40) integrated various transcriptomic data with computational systems biology and machine learning algorithms and identified 52 common drug targets, including PPARγ, for COVID-19 treatment (40).

**Figure 4:**
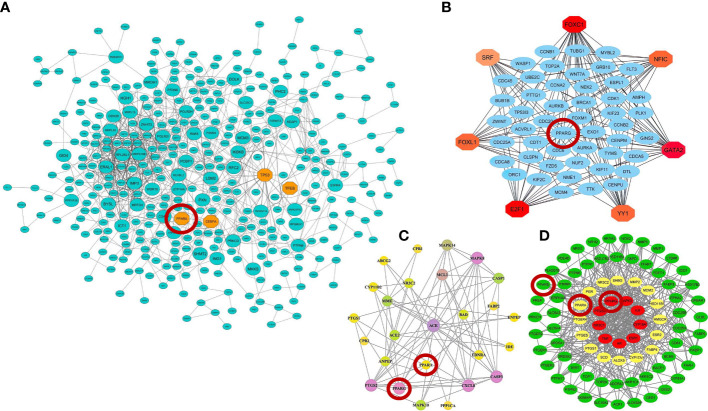
Protein–protein interaction (PPI) networks of therapeutic candidate modules for COVID-19 therapy obtained by **(A)** our group (39) **(B)** Auwul et al. (40), **(C)** Oh et al. (183), and **(D)** Li et al. (185). These modules had the most biological associations with the immunopathogenesis of COVID-19. Large circles represent hub-high traffic genes. PPAR genes as potential therapeutic targets for COVID-19 pandemic are highlighted by red circles in these PPI networks.

Moreover, further studies using pharmacological network approaches have identified PPARα and PPARγ as promising drug/therapeutic targets to control inflammation caused by host–SARS-CoV-2 interactions (183, 184). On the other hand, recently, a study introduced glycyrrhetinic acid as an essential drug against cytokine storm in COVID-19 patients (185). During this study, using protein–protein interaction (PPI) network and molecular docking techniques, it was well established that glycyrrhetinic acid activates or represses 84 core genes to counter the cytokine storm during COVID-19 using multiple strategies (185). As an important result of this study, one of these glycyrrhetinic acid strategies to deal with the cytokine storm was to target PPARγ, PPARα, and PPARβ/δ for activation (185). **Figure 4** shows that the PPI structure of the candidate modules identified by these studies that contained critical therapeutic targets, including PPARα, PPARβ/δ, and PPARγ for COVID-19 therapy.

Interestingly, the use of PPARs agonists to activate them to repress the inflammatory processes during COVID-19 has recently attracted much attention. In this regard, it has been demonstrated that PPAR activation through synthetic and nutritional compounds can be an efficient management program to overcome the cytokine storm and prevent the deleterious inflammatory effects after coronavirus infection (38, 186). Moreover, a recent study suggested using synthetic and natural ligands of PPARs in order to target NF-κB transcriptional activity and reduce inflammatory response as an attractive strategy for managing the nutrition of COVID-19 patients (187). Following these results, the recent literature also reported that several natural and synthetic PPARγ agonists suppress NF-κB activity through PPARγ activation, leading to reduced levels of proinflammatory cytokines such as IL1β, IL6, TNF-α, IL18, IFN-γ, IL8, and IL12 (38). It has been well established that PPARα activation using oleoylethanolamide, in addition to suppressing TLR4-mediated NF-κB signaling cascade and reducing proinflammatory cytokines such as COX2, IL6, CRP, IL1β, TNF-α, and iNOS, is also associated with increased levels of anti-inflammatory factors such as IL10 (56, 188–190). Additionally, it has recently been shown that fenofibrate is a PPARα agonist with anti-inflammatory, anti-oxidant, and anti-thrombotic properties that exerts broad anti-inflammatory effects such as inhibition of iNOS, repression of COX2 and MMP9, activation of inhibitory kappa B (IκB), and release of adiponectin through the activating of PPARα during SARS-CoV-2 infection (150). Furthermore, previous studies reported that fenofibrate inhibits viral replication in lung epithelial cells by reversing the metabolic changes caused by SARS-CoV-2 (175). On the other hand, natural astaxanthin (ASX), an important PPARγ agonist, has a clinically proven safety profile with anti-oxidant, anti-inflammatory, and immunomodulatory properties. Interestingly, mounting evidence from clinical studies suggests that this PPAR agonist prevents the ARDS/ALI in COVID-19 patients by downregulating NF-κB and JAK/STAT signaling and then reducing TNF-α, IL1β, and IL6 levels. Moreover, ASX caused a change in the inflammatory response of Th1 cells to Th2, leading to a shift from proinflammatory cytokine secretion to anti-inflammatory cytokine secretion (191). ASX also exerts an anti-oxidant effect and prevents oxidative damage through (1) inhibition of NLRP3 inflammasome and HIF1-α, and (2) suppression of plasma CRP, iNOS, COX2, PGE2, and ICAM1, respectively. Accordingly, ASX-mediated activation of PPARγ has been proposed as an effective therapeutic strategy to control host inflammatory and immune responses, antagonize the cytokine storm, and prevent deleterious inflammatory effects following COVID-19 (191). Moreover, troglitazone, an insulin-sensitizing drug that is prescribed for treating type 2 diabetes mellitus (192), is a synthetic PPARγ agonist that interferes with NF-κB activity and exerts its anti-inflammatory effects through the activation of PPARγ (38). Interestingly, this drug has been introduced as one of the most suitable options for applying anti-inflammatory effects for COVID-19-inducted hyperinflammation (38). In addition to troglitazone, pioglitazone (a synthetic agonist of PPARγ, see **Table 1**) is another member of the thiazolidinedione (TZD) family that has significant anti-inflammatory effects and has been suggested by Carboni et al. (193) as a support drug for the reduction in many inflammatory parameters in COVID-19 patients (193). Additionally, pioglitazone can reduce SARS-CoV-2 RNA synthesis and replication through potential inhibition of 3-chymotrypsin-like protease (3CL-Pro) (194). Moreover, it has recently been suggested that activation of PPARγ by agonists such as cannabidiol reduces cytokine secretion, pulmonary inflammation, and fibrosis in the lung of the patients during SARS-CoV-2 infection (195). Intriguingly, zinc supplementation has also been reported to have potential health benefits for managing inflammation in COVID-19 by suppressing the expression of many cytokines and adhesion molecules through increasing the expression of PPARα (196). Furthermore, natural compounds such as gamma-oryzanol (the main bioactive constituent from rice bran and germ) have been introduced as a possible adjunctive therapy to prevent the cytokine storm in COVID-19 patients, as this compound positively increases the expression of PPARγ in adipose tissue and as a result reduces the levels of inflammatory cytokines including TNF-α, IL6, and MCP1 (157). To the best of our knowledge, no information is yet available on the role of PPARβ/δ agonists during SARS-CoV-2 infection. Therefore, future research should investigate the anti-inflammatory effects of natural and synthetic agonists of PPARβ/δ during the COVID-19 pandemic. However, looking at the previous literature on similar inflammatory lung diseases in humans, it can be concluded that PPARβ/δ agonists have significant anti-inflammatory effects during lung infection (197). Conversely, using existing natural and synthetic ligands of PPARs may have limitations or challenges. For instance, recent data show that using natural and synthetic ligands of PPARs is highly dose-dependent and can interact with non-PPAR targets due to the complexities of the drug–target complex (94). Moreover, it has been reported that some ligands of PPARs can exert selective agonistic or antagonistic regulatory effects depending on the cell context (191, 198, 199). Additionally, it has been highlighted that using synthetic agonists of PPARs can lead to serious clinical complications, including bone fracture, heart failure, cardiovascular risk, liver failure, gastrointestinal bleeding, and liver and kidney toxicity (200).

These findings highlight the potential role of PPAR subtypes (particularly PPARγ) and their ligands with anti-inflammatory effects during the COVID-19 pandemic, which can be promising candidates for inhibiting key inflammatory factors (especially NF-κB and AP-1), thus regulating inflammation during SARS-CoV-2 infection. Therefore, any intervention methods aimed at activating/upregulating/overexpressing of PPAR subtypes could be a promising therapeutic strategy to reduce the hyperinflammatory response in COVID-19 patients and prevent the cytokine storm.

## 6 Conclusions and future prospects

COVID-19 is an emerging global health threat caused by SARS-CoV-2 infection with severe inflammatory complications. Treatment of severely ill patients is an important healthcare issue. Despite developing various vaccines for disease prevention, there is still no definitive treatment solution for COVID-19 patients. The massive cytokine secretion caused by the exacerbation of the host inflammatory system in response to SARS-CoV-2 infection is known as a “cytokine storm”, which is directly related to the progression of the disease from mild to severe. In recent years, among previous efforts, it has been suggested that one of the most effective strategies for improving the survival of COVID-19 patients and reducing the severity of the disease is to control the hyperinflammatory response and interfere with cytokine storm. Cytokine storm has been one of the main characteristics of disease severity, decreased lung function, ARDS, ALI, and MOF, and ultimately, the death of COVID-19 patients. Therefore, paying attention to anti-inflammatory factors and examining their response during SARS-CoV-2 infection can provide the basic solution to deal with COVID-19-inducted cytokine storm.

PPARs are ligand-dependent transcription factors belonging to the nuclear receptor superfamily, which are the main regulators of lipid and glucose metabolism. This transcription factor family consists of three subtypes: PPARα, PPARβ/δ, and PPARγ. In the last decade, it has been well established that these subtypes, in addition to their central role in metabolism and energy balance, play important roles in cell proliferation, differentiation, the immune cell system, and inflammation. Concerning inflammation and inflammation-related disease, PPARs play an important anti-inflammatory role as critical inhibitors of the host inflammatory response through adverse regulatory effects on active inflammatory transcription factors such as NF-κB, AP-1, NFAT, and STATs. Currently, extensive clinical and omics studies indicate downregulation in the expression of PPARs in response to SARS-CoV-2 infection, which has been proposed as one of the main causes of SARS-CoV-2 immunopathogenesis to exacerbate the host inflammatory response.

On the other hand, it has been highlighted that the activation of PPARs through natural and synthetic ligands is associated with the reduction of hyperinflammatory response, prevention of cytokine storm, and reduction in disease severity in COVID-19 patients. Therefore, this makes them attractive and practical targets for developing novel therapeutic strategies against COVID-19 and cytokine storm. However, the previous literature has indicated that using existing natural and synthetic ligands of PPARs may lead to severe clinical complications.

Therefore, considering the anti-inflammatory importance of PPARs to control the hyperinflammatory response during COVID-19, further research should deeply investigate the individual or collective effects of PPAR subtypes to inhibit cytokine storms during SARS-CoV-2 infection. Moreover, considering the side-effects and challenges of using existing natural and synthetic ligands to activate PPARs, further exploration of the underlying mechanisms is needed to establish new pathways of PPARs activation without causing severe clinical side-effects.

## Author contributions

AH and AB conceived the ideas. AH, AB, AS, and BK designed the study. AH, AB, AS, BT-F, and SI determined the methodology. AH, AB, BK, FG, AS, AA, and MA validated and interpreted the results. AH, AB, BT-F, AS, MA, FG, and SI investigated and collected resources. AH, AB, and BK wrote and prepared the original draft. AH, AB, AS, AA, and BK reviewed and edited the manuscript. All authors contributed to the article and approved the submitted version.
