# Corrigendum: The role of peroxisome proliferator-activated receptors in the modulation of hyperinflammation induced by SARS-CoV-2 infection: A perspective for COVID-19 therapy

**DOI:** 10.3389/fimmu.2024.1422261

**Published:** 2024-05-08

**Authors:** Aliakbar Hasankhani, Abolfazl Bahrami, Bahareh Tavakoli-Far, Setare Iranshahi, Farnaz Ghaemi, Majid Reza Akbarizadeh, Ali H. Amin, Bahman Abedi Kiasari, Alireza Mohammadzadeh Shabestari

**Affiliations:** ^1^ Department of Animal Science, College of Agriculture and Natural Resources, University of Tehran, Karaj, Iran; ^2^ Faculty of Agricultural Sciences and Engineering, University of Tehran, Karaj, Iran; ^3^ Dietary Supplements and Probiotic Research Center, Alborz University of Medical Sciences, Karaj, Iran; ^4^ Department of Physiology and Pharmacology, School of Medicine, Alborz University of Medical Sciences, Karaj, Iran; ^5^ School of Pharmacy, Shahid Beheshty University of Medical Sciences, Tehran, Iran; ^6^ Department of Biochemistry, Faculty of Advanced Sciences and Technology, Tehran Medical Sciences, Islamic Azad University, Tehran, Iran; ^7^ Department of Pediatric, School of Medicine, Amir al momenin Hospital, Zabol University of Medical Sciences, Zabol, Iran; ^8^ Zoology Department, Faculty of Science, Mansoura University, Mansoura, Egypt; ^9^ Virology Department, Faculty of Veterinary Medicine, University of Tehran, Tehran, Iran; ^10^ Department of Dental Surgery, Mashhad University of Medical Sciences, Mashhad, Iran; ^11^ Khorasan Covid-19 Scientific Committee, Mashhad, Iran

**Keywords:** SARS-CoV-2, cytokine storm, PPARs, hyperinflammation, COVID-19 therapy

**Incorrect Affiliation**

In the published article, there was an error in affiliation 2. Instead of “Biomedical Center for Systems Biology Science Munich, Ludwig-Maximilians-University, Munich, Germany”, it should be “Faculty of Agricultural Sciences and Engineering, University of Tehran, Karaj, Iran”.

The authors apologize for this error and state that this does not change the scientific conclusions of the article in any way. The original article has been updated.

